# Subtype-Specific Prognostic and Molecular Significance of ADGRD1 in Lung Adenocarcinoma and Lung Squamous Cell Carcinoma

**DOI:** 10.3390/cimb48090927

**Published:** 2026-09-10

**Authors:** Su-Min Ryu, Guen-Hye Kim, Yunah Nam, Jun-Chae Lee, Jae-Ho Lee, Hyowon Hong

**Affiliations:** 1 Medical Course, School of Medicine, Keimyung University, Daegu 42601, Republic of Korea; suminy2150@gmail.com (S.-M.R.); kkkh4468@naver.com (G.-H.K.); med.yunahnam@gmail.com (Y.N.); chai1703@naver.com (J.-C.L.); 2 Department of Anatomy, School of Medicine, Keimyung University, Daegu 42601, Republic of Korea

**Keywords:** ADGRD1, lung squamous cell carcinoma, non-small cell lung cancer, TCGA

## Abstract

Adhesion G protein-coupled receptor D1 (ADGRD1), formerly known as GPR133, has been implicated in tumor-associated signaling; however, its histology-specific clinicopathologic and prognostic significance in non-small cell lung cancer (NSCLC) remains incompletely characterized. Clinical and survival data from The Cancer Genome Atlas lung adenocarcinoma (TCGA-LUAD) and lung squamous cell carcinoma (TCGA-LUSC) cohorts were integrated with harmonized gene-expression data obtained from the Genomic Data Commons (GDC). ADGRD1 expression was dichotomized using a cohort-specific median cutoff and additionally analyzed as a continuous standardized variable. Clinicopathologic associations, Pearson correlations with prespecified subtype-associated genes, Kaplan–Meier survival analyses, and univariate and multivariable Cox proportional hazards regression analyses were performed. Benjamini–Hochberg false-discovery-rate correction was applied to molecular correlation analyses. Independent validation was performed using GSE30219 and GSE50081, followed by random-effects meta-analysis and proportional hazards sensitivity analyses. High ADGRD1 expression was associated with favorable overall survival (OS) in LUAD in both univariate (HR = 0.542, 95% CI = 0.400–0.734, *p* < 0.001) and clinically adjusted Cox analyses (HR = 0.543, 95% CI = 0.398–0.740, *p* < 0.001). Continuous-expression analyses showed concordant results, and the association remained significant in an additional LUAD model incorporating EGFR mutation status (HR = 0.611, 95% CI = 0.391–0.956, *p* = 0.031). High ADGRD1 expression was also associated with favorable recurrence-free survival (RFS) in LUAD. In contrast, high ADGRD1 expression was associated with adverse OS in LUSC in both univariate (HR = 1.444, 95% CI = 1.099–1.898, *p* = 0.008) and clinically adjusted analyses (HR = 1.501, 95% CI = 1.137–1.982, *p* = 0.004), whereas RFS was not significantly different. After false-discovery-rate correction, ADGRD1 correlated positively with TP53 expression in LUAD and inversely with CDKN2A, SOX2, and PIK3CA in LUSC. Independent GEO validation supported the favorable LUAD association (pooled HR = 0.675, 95% CI = 0.538–0.847, *p* < 0.001), whereas the adverse LUSC association was not independently reproduced (pooled HR = 0.909, 95% CI = 0.686–1.204, *p* = 0.504). ADGRD1 shows histology-dependent prognostic associations in NSCLC. Its favorable association with survival in LUAD was robust across dichotomized and continuous analyses, multivariable adjustment, EGFR-mutation sensitivity analysis, and independent GEO cohorts. In contrast, the adverse association observed in TCGA-LUSC was not reproduced externally. These findings support further evaluation of ADGRD1 as a subtype-specific prognostic marker, particularly in LUAD.

## 1. Introduction

Non-small cell lung cancer (NSCLC) comprises a heterogeneous group of malignancies with substantial histologic, molecular, and clinical diversity [1,2]. Lung adenocarcinoma (LUAD) and lung squamous cell carcinoma (LUSC), the two major NSCLC histologies, exhibit distinct genomic and transcriptomic landscapes [3,4]. LUAD frequently harbors alterations affecting receptor tyrosine kinase and RAS–RAF signaling, including EGFR, KRAS, BRAF, and ALK, whereas LUSC is characterized by frequent TP53 disruption, CDKN2A alterations, SOX2 amplification, and abnormalities affecting PI3K-related signaling [3,4,5,6]. Consequently, the biological and prognostic significance of an individual molecular marker may differ according to histologic context rather than being uniform across NSCLC [3,4,5].

ADGRD1, formerly designated GPR133, belongs to the adhesion G protein-coupled receptor (aGPCR) family, a structurally distinct class of receptors characterized by large extracellular regions combined with seven-transmembrane signaling domains [7,8,9,10]. Adhesion GPCRs participate in cell–cell and cell–matrix interactions, mechanosensing, migration, and intracellular signaling and have increasingly been implicated in cancer-associated biological processes [9,11,12]. Experimental studies have demonstrated that ADGRD1 supports glioblastoma growth and that its activity can be influenced by hypoxic conditions, illustrating the context-dependent biological functions of this receptor [13,14].

ADGRD1 has also been investigated in NSCLC. Lv et al. reported associations between ADGRD1 expression, prognosis, and immune-related characteristics and described different prognostic patterns between LUAD and LUSC [15]. Thus, the existence of histology-dependent survival associations is not itself a novel observation [15]. However, the reproducibility of these associations across independent cohorts, their robustness to clinical adjustment and continuous-expression modeling, and the relationship between ADGRD1 and representative subtype-associated transcriptional profiles remain incompletely characterized [15]. Recent subtype-specific transcriptomic investigations have further demonstrated the importance of evaluating LUAD and LUSC separately when assessing the clinical relevance of candidate molecular markers [16,17].

Accordingly, the present study investigated the clinicopathologic, molecular, and prognostic significance of ADGRD1 separately in LUAD and LUSC using TCGA-derived clinical data integrated with harmonized GDC gene-expression measurements [3,4]. In addition to clinicopathologic and survival analyses, we evaluated correlations between ADGRD1 and prespecified subtype-associated genes, tested the robustness of prognostic associations using both median-dichotomized and standardized continuous expression, and performed independent validation in GSE30219 and GSE50081 [18,19]. This integrated approach was designed to distinguish reproducible histology-specific associations from findings that may be dependent on an individual cohort or analytical cutoff.

## 2. Materials and Methods

### 2.1. TCGA Clinical Data and GDC Gene-Expression Integration

Clinical and survival information from TCGA-LUAD and TCGA-LUSC was obtained from investigator-curated TCGA-derived analytic datasets supplied in SPSS format [3,4]. TCGA patient identifiers were extracted from the sample identifiers and matched to cases in the National Cancer Institute Genomic Data Commons (GDC) [20]. One duplicated LUAD patient identifier was resolved so that each unique TCGA patient contributed a single analytic record. Following patient-level matching, ADGRD1 expression data were available for 493 unique LUAD patients and 489 LUSC patients.

Harmonized gene-expression values were retrieved from the GDC gene-expression resource for ADGRD1 and all prespecified molecular genes used in the correlation analyses [20]. FPKM upper-quartile normalized values (FPKM-UQ) were transformed as log2(FPKM-UQ + 1) before statistical analysis. All genes included in the correlation analysis were therefore obtained using the same expression source and normalization procedure.

Patients were classified into ADGRD1-high and ADGRD1-low groups independently within LUAD and LUSC using the cohort-specific median ADGRD1 expression value. The median cutoff was prespecified to avoid selection of an outcome-optimized threshold. To evaluate whether the prognostic findings depended on dichotomization, ADGRD1 expression was additionally standardized within each histologic cohort and analyzed continuously per one-standard-deviation (SD) increase.

Clinical variables included age, sex, smoking status, pathologic T category, pathologic N category, pathologic M category, and overall pathologic stage when available. Missing clinical values were not imputed, and available-case analysis was used for individual clinicopathologic comparisons.

Overall survival (OS) was defined using the OS status and follow-up variables contained in the curated clinical files. The investigator-provided files additionally contained pre-existing variables designated RFS and RFS.time, which were used for recurrence-free survival (RFS) analyses. The present study did not independently reconstruct this recurrence endpoint from raw TCGA clinical data. This distinction was retained because standardized TCGA clinical resources separately define OS, disease-free interval, disease-specific survival, and progression-free interval [21].

### 2.2. Clinicopathologic Association Analysis

Associations between median-dichotomized ADGRD1 expression and categorical clinicopathologic variables were assessed separately in LUAD and LUSC. Pearson’s chi-square test was applied when expected cell-count assumptions were adequately satisfied. Fisher’s exact test was used for sparse 2 × 2 contingency tables, whereas the Fisher–Freeman–Halton approach with Monte Carlo estimation was used for larger sparse contingency tables. Two-sided *p* values < 0.05 were considered statistically significant.

Verified EGFR mutation status was additionally evaluated in the LUAD cohort when mutation-positive versus wild-type information was available. The supplied LUSC analytic file contained information concerning whether EGFR testing was performed but did not provide a verified mutation-positive versus wild-type variable; consequently, EGFR mutation status was not analyzed as a clinicopathologic characteristic in LUSC.

### 2.3. Molecular Correlation Analysis

Representative molecular genes were prespecified according to the established molecular characteristics of the two histologic subtypes [3,4,6]. The LUAD analysis included EGFR, KRAS, TP53, ALK, and BRAF, whereas the LUSC analysis included TP53, CDKN2A, SOX2, PIK3CA, and NOTCH1 [3,4,6].

Pearson correlation coefficients were calculated between continuous log2(FPKM-UQ + 1)-transformed ADGRD1 expression and the expression of each prespecified gene using pairwise complete observations. The correlation analyses were performed in R using the cor.test() function with method = “pearson” in the stats package. Because five prespecified molecular correlations were evaluated within each histologic subtype, Benjamini–Hochberg false-discovery-rate (FDR) correction was applied independently in LUAD and LUSC using the p.adjust() function with method = “BH”. An FDR-adjusted q value < 0.05 was considered statistically significant after correction for multiple testing.

The purpose of this analysis was to characterize the transcript-level molecular context associated with ADGRD1 expression rather than to evaluate mutation co-occurrence or infer direct regulatory relationships. Accordingly, all correlations were based on continuous gene-expression measurements obtained from the same harmonized GDC expression source.

### 2.4. Kaplan–Meier and Cox Regression Analyses

Kaplan–Meier survival curves and log-rank tests were used to compare overall survival (OS) and recurrence-free survival (RFS) between the ADGRD1-high and ADGRD1-low groups. Numbers at risk were displayed beneath the survival curves. Kaplan–Meier analyses were considered unadjusted survival comparisons, whereas adjustment for potential clinicopathologic confounding was performed using Cox proportional hazards regression.

Univariate Cox proportional hazards regression was first used to estimate the association between the ADGRD1 expression group and OS. The primary multivariable Cox models were fitted separately in LUAD and LUSC and included ADGRD1 expression group, age group, sex, smoking status, and pathologic stage. Pathologic stage was entered as an ordinal clinical covariate. Hazard ratios (HRs) with 95% confidence intervals (CIs) are reported.

To reduce dependence on the cohort-specific median cutoff, corresponding Cox regression analyses were additionally performed using standardized continuous ADGRD1 expression, with HRs estimated per one-standard-deviation (SD) increase. In LUAD, an additional sensitivity model incorporated verified EGFR mutation status together with ADGRD1 expression group, age group, sex, smoking status, and pathologic stage. Because verified EGFR mutation information was available only for a subset of LUAD patients, this model was treated as a sensitivity analysis rather than as the primary multivariable model.

Treatment history, performance status, comorbidities, and other potentially relevant prognostic factors were not consistently available in the curated analytic datasets and therefore could not be incorporated into the multivariable models. The possibility of residual confounding was consequently considered when interpreting the adjusted survival associations.

The proportional hazards assumption for the primary multivariable TCGA models was evaluated using Schoenfeld residual-based testing. TCGA/GDC data integration and statistical analyses were conducted in R version 4.5.3. Data import, processing, and GDC application programming interface (API) queries were performed primarily using the haven, dplyr, tidyr, purrr, stringr, httr, jsonlite, readr, and tibble packages. Cox proportional hazards regression and proportional hazards diagnostics were performed using the survival package, and model estimates were extracted and summarized using broom. All statistical tests were two-sided, and *p* values < 0.05 were considered statistically significant.

### 2.5. Independent GEO Validation

Independent validation was performed using the publicly available GSE30219 and GSE50081 NSCLC cohorts [18,19]. Both datasets were generated using the Affymetrix Human Genome U133 Plus 2.0 Array platform. ADGRD1 expression was represented by probe 232267_at, and analyses were conducted separately according to LUAD and LUSC histology.

For Kaplan–Meier validation, patients were divided using the cohort- and subtype-specific median ADGRD1 expression level, and OS was compared using the log-rank test. For quantitative external validation, ADGRD1 expression was standardized independently within each cohort and histologic subtype, and Cox regression was performed per one-SD increase in expression.

The adjusted GSE30219 models incorporated age, sex, T stage, and N stage. The adjusted GSE50081 LUAD model included age, sex, and pathologic stage. An adequately specified adjusted model was not estimated for GSE50081 LUSC because of the limited sample size and available clinical covariate structure.

Unadjusted continuous-expression HRs from GSE30219 and GSE50081 were pooled separately for LUAD and LUSC using random-effects meta-analysis with restricted maximum likelihood estimation. The proportional hazards assumption was evaluated using Schoenfeld residuals. Because evidence of non-proportional hazards was detected for ADGRD1 in GSE30219 LUAD, additional analyses included a time-varying coefficient model using an ADGRD1 × log(1 + time/12) interaction and a 60-month restricted mean survival time (RMST) analysis. External-validation analyses were conducted in R version 4.5.3. GEO datasets were retrieved and processed using GEOquery and Biobase, and probe annotation was verified using AnnotationDbi and hgu133plus2.db. Survival analyses and Kaplan–Meier visualization were performed using survival and survminer, respectively, and a random-effects meta-analysis was performed using metafor. The 60-month RMST sensitivity analysis was performed using survRM2.

## 3. Results

### 3.1. Clinicopathologic Characteristics According to ADGRD1 Expression

Following patient-level integration of the clinical and GDC expression datasets, ADGRD1 expression data were available for 493 unique LUAD patients and 489 LUSC patients. Median dichotomization yielded 247 ADGRD1-low and 246 ADGRD1-high patients in LUAD and 245 ADGRD1-low and 244 ADGRD1-high patients in LUSC.

In LUAD, ADGRD1 expression was significantly associated with overall pathologic stage (*p* = 0.043), whereas N stage was not significantly associated with ADGRD1 expression (*p* = 0.075) (Table S1).

In LUSC, ADGRD1 expression was significantly associated with sex (*p* = 0.023), whereas T stage did not reach statistical significance (*p* = 0.067) (Table S1).

### 3.2. Survival and Cox Regression Analyses

**Figure 1:**
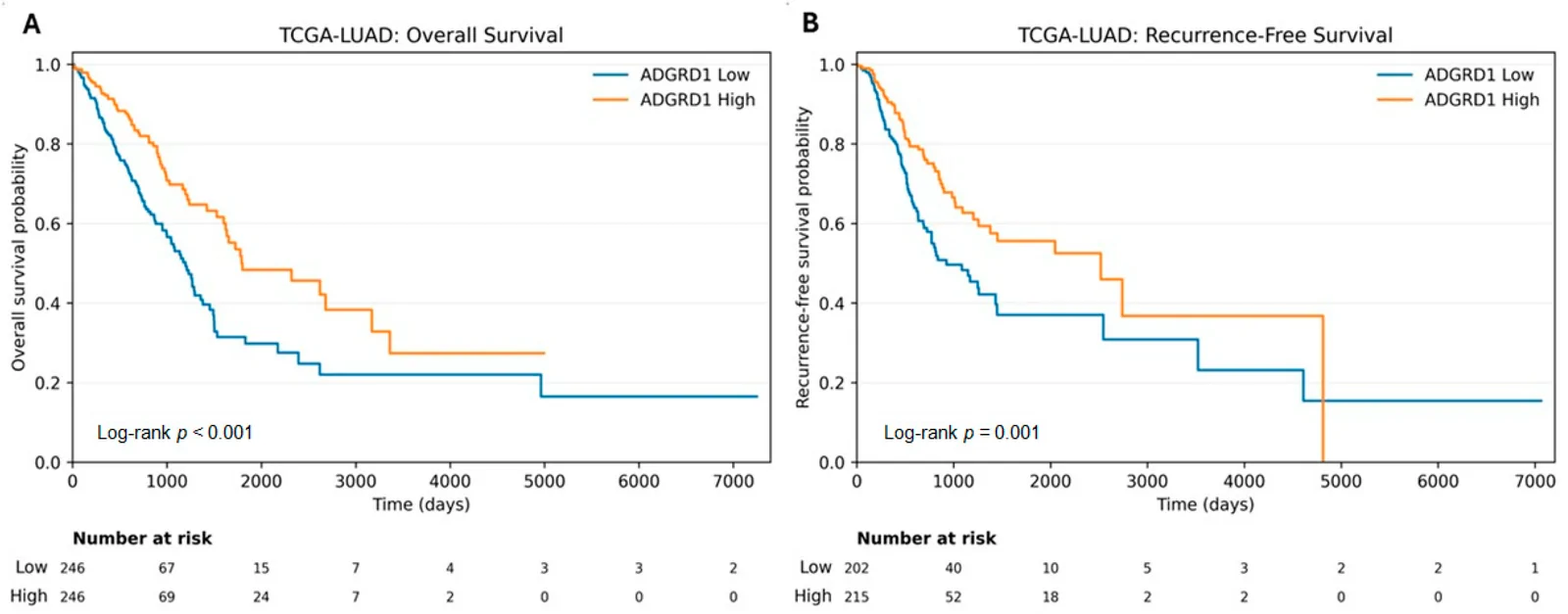
Kaplan–Meier survival analyses according to ADGRD1 expression in TCGA-LUAD: (**A**) Overall survival and (**B**) recurrence-free survival according to cohort-specific median ADGRD1 expression. *p* values were calculated using the log-rank test. Numbers at risk are shown below the survival curves.

OS information was available for 492 LUAD patients, including 179 deaths. Patients with high ADGRD1 expression demonstrated significantly better OS than those with low expression (log-rank *p* < 0.001) (Figure 1A). RFS information was available for 417 LUAD patients, including 145 recurrence events, and high ADGRD1 expression was similarly associated with more favorable RFS (log-rank *p* = 0.001) (Figure 1B).

**Figure 2:**
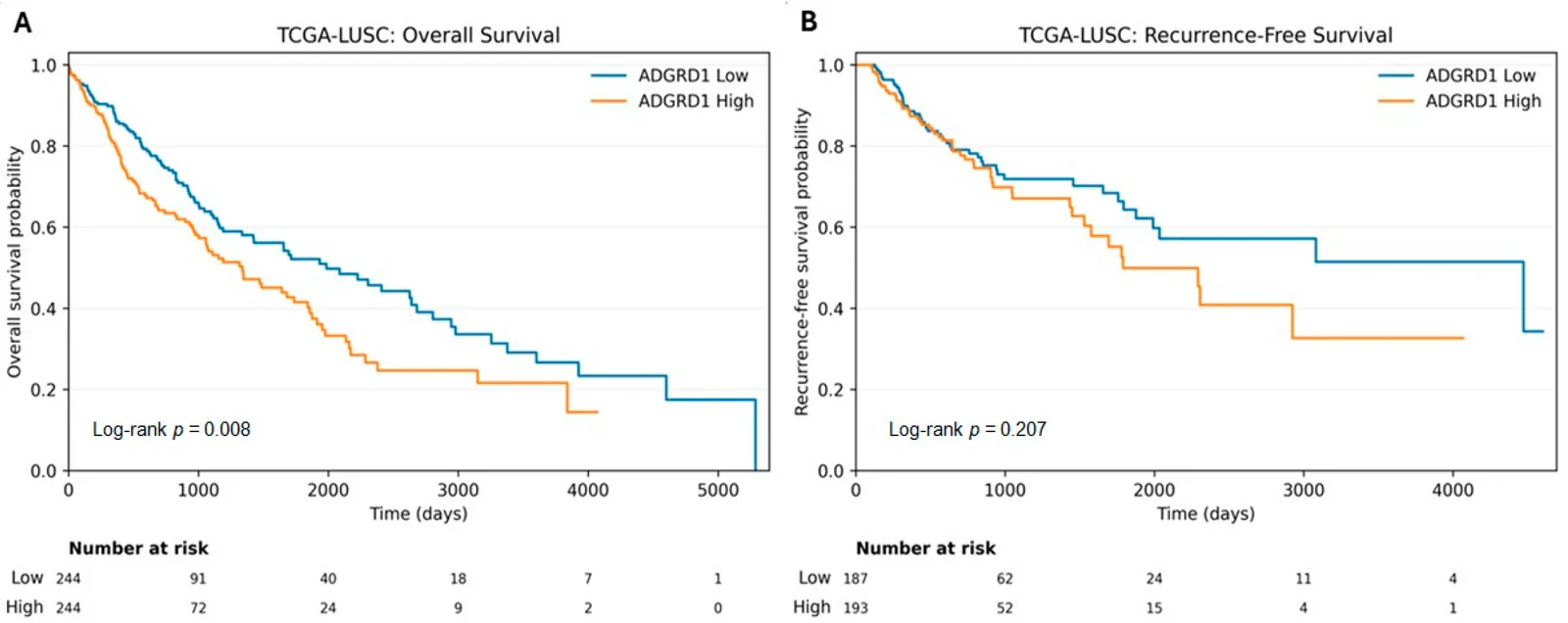
Kaplan–Meier survival analyses according to ADGRD1 expression in TCGA-LUSC: (**A**) Overall survival and (**B**) recurrence-free survival according to cohort-specific median ADGRD1 expression. *p* values were calculated using the log-rank test. Numbers at risk are shown below the survival curves.

In LUSC, OS information was available for 488 patients, including 212 deaths. High ADGRD1 expression was associated with significantly poorer OS (log-rank *p* = 0.008) (Figure 2A). RFS was available for 380 patients, including 99 events, but the difference between expression groups was not statistically significant (log-rank *p* = 0.207) (Figure 2B).

In LUAD, high ADGRD1 expression was associated with a significantly lower risk of death in univariate Cox analysis (HR = 0.542, 95% CI = 0.400–0.734, *p* < 0.001) (Table 1). The association remained significant after adjustment for age group, sex, smoking status, and pathologic stage (HR = 0.543, 95% CI = 0.398–0.740, *p* < 0.001). Continuous-expression analysis provided concordant evidence, with an HR of 0.780 per one-SD increase in the unadjusted model (95% CI = 0.668–0.909, *p* = 0.002) and an HR of 0.787 after clinical adjustment (95% CI = 0.673–0.919, *p* = 0.002).

The favorable LUAD association remained significant in the additional EGFR-mutation sensitivity model. Among 209 complete cases with 88 deaths, high ADGRD1 expression was associated with a reduced risk of death after adjustment for age group, sex, smoking status, pathologic stage, and verified EGFR mutation status (HR = 0.611, 95% CI = 0.391–0.956, *p* = 0.031).

In LUSC, high ADGRD1 expression was associated with an increased risk of death in univariate analysis (HR = 1.444, 95% CI = 1.099–1.898, *p* = 0.008) (Table 1). This adverse association remained significant after clinical adjustment (HR = 1.501, 95% CI = 1.137–1.982, *p* = 0.004). Continuous-expression analyses were concordant, with HRs of 1.183 per one-SD increase in the unadjusted model (95% CI = 1.041–1.345, *p* = 0.010) and 1.206 in the clinically adjusted model (95% CI = 1.062–1.369, *p* = 0.004).

Schoenfeld residual testing did not demonstrate a significant violation of the proportional hazards assumption for ADGRD1 in the primary TCGA clinically adjusted models in LUAD (*p* = 0.137) or LUSC (*p* = 0.639).

### 3.3. Correlation of ADGRD1 Expression with Cancer-Related Gene Expression

In LUAD, ADGRD1 expression was positively correlated with TP53 expression (r = 0.152, *p* < 0.001, q = 0.004), and this association remained significant after FDR correction (Table 2). A weak positive correlation with BRAF was nominally significant (r = 0.092, *p* = 0.041) but did not remain significant after FDR correction (q = 0.103). No significant associations were observed with EGFR (r = −0.067, *p* = 0.140, q = 0.233), KRAS (r = −0.011, *p* = 0.800, q = 0.800), or ALK (r = −0.037, *p* = 0.409, q = 0.512).

In LUSC, significant inverse correlations were identified between ADGRD1 and CDKN2A (r = −0.119, *p* = 0.008, q = 0.014), SOX2 (r = −0.247, *p* < 0.001, q < 0.001), and PIK3CA (r = −0.190, *p* < 0.001, q < 0.001) (Table 2). Correlations with TP53 (r = −0.047, *p* = 0.299, q = 0.374) and NOTCH1 (r ≈ 0.000, *p* = 0.993, q = 0.993) were not statistically significant. Although several associations remained significant after correction, their magnitudes were modest and should therefore be regarded as transcript-level associations rather than evidence of direct molecular regulation.

### 3.4. Independent External Validation

**Figure 3:**
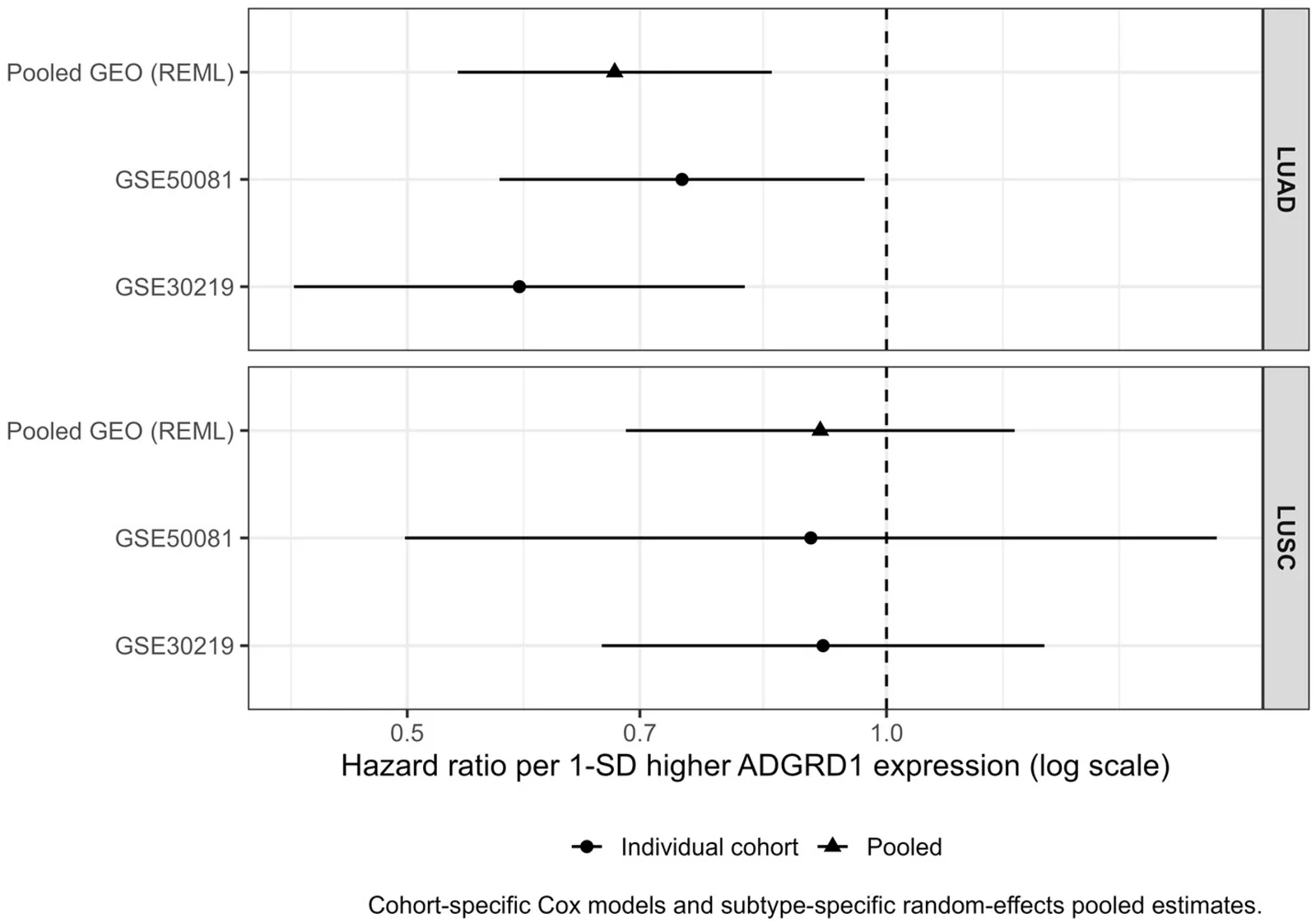
Independent external validation of the prognostic association of ADGRD1 expression in LUAD and LUSC. Forest plots show unadjusted cohort-specific hazard ratios for overall survival per one-standard-deviation increase in ADGRD1 expression in GSE30219 and GSE50081 together with subtype-specific random-effects pooled estimates. An HR below 1 indicates a favorable association with higher ADGRD1 expression.

The favorable prognostic association observed in TCGA-LUAD was supported by the independent GEO cohorts (Table 3 and Figure 3). Kaplan–Meier analyses using cohort- and subtype-specific median expression cutoffs showed a similar pattern in the independent GEO cohorts (Figure S1). In GSE30219 LUAD, higher ADGRD1 expression was associated with better OS in the unadjusted continuous-expression model (HR = 0.588, 95% CI = 0.424–0.815, *p* = 0.001) and after adjustment for age, sex, T stage, and N stage (HR = 0.559, 95% CI = 0.401–0.780, *p* < 0.001). In GSE50081 LUAD, the unadjusted association was also significant (HR = 0.744, 95% CI = 0.571–0.969, *p* = 0.028), whereas the adjusted estimate retained the same favorable direction but did not reach statistical significance (HR = 0.790, 95% CI = 0.607–1.028, *p* = 0.079).

Random-effects meta-analysis of the two LUAD cohorts demonstrated a significant pooled association between higher ADGRD1 expression and favorable OS (HR = 0.675, 95% CI = 0.538–0.847, *p* < 0.001) (Figure 3).

In contrast, the adverse association observed in TCGA-LUSC was not independently reproduced. Consistent with the continuous-expression analyses, Kaplan–Meier analyses did not show a significant survival difference between the ADGRD1-high and ADGRD1-low groups in either LUSC cohort (Figure S1). Continuous ADGRD1 expression was not significantly associated with OS in GSE30219 LUSC (HR = 0.912, 95% CI = 0.662–1.257, *p* = 0.574) or GSE50081 LUSC (HR = 0.896, 95% CI = 0.498–1.612, *p* = 0.715). The pooled LUSC estimate was likewise nonsignificant (HR = 0.909, 95% CI = 0.686–1.204, *p* = 0.504).

The proportional hazards assumption was not satisfied for ADGRD1 in the standard GSE30219 LUAD continuous Cox model (Schoenfeld *p* = 0.027) (Table S2). Time-varying analysis indicated that the favorable association was strongest during earlier follow-up, with adjusted HRs of 0.374 at 12 months, 0.544 at 36 months, and 0.677 at 60 months (Figure S2). Complementary 60-month RMST analysis showed an estimated 14.98-month survival advantage for the ADGRD1-high group compared with the low-expression group (95% CI = 7.46–22.50 months, *p* < 0.001).

## 4. Discussion

The principal finding of the present study is that the prognostic association of ADGRD1 differs according to NSCLC histology. Higher ADGRD1 expression was associated with favorable OS and RFS in LUAD, whereas higher expression was associated with adverse OS in the TCGA-LUSC cohort. Histology-dependent prognostic patterns have previously been reported for ADGRD1 in NSCLC [15]; therefore, the principal contribution of the present study is not the initial identification of an opposite prognostic pattern but its systematic evaluation using harmonized expression data, clinical adjustment, continuous-expression sensitivity analysis, multiple-testing-corrected molecular correlations, and independent external validation [15].

The LUAD findings were particularly robust. High ADGRD1 expression was associated with a substantially reduced mortality risk in the median-dichotomized analysis, and the magnitude of the association changed minimally following adjustment for age, sex, smoking status, and pathologic stage. Importantly, standardized continuous-expression analyses yielded the same favorable direction, indicating that the association was not dependent on the median cutoff. Furthermore, the association remained statistically significant after EGFR mutation status was incorporated into a sensitivity model, despite the considerably smaller complete-case sample. Together, these findings support an association between ADGRD1 expression and a favorable LUAD prognostic phenotype that cannot readily be explained by the measured clinical characteristics alone.

Independent validation provided further support for this interpretation. Both GSE30219 and GSE50081 showed favorable continuous-expression HRs in LUAD, and the random-effects pooled estimate was statistically significant. The association therefore remained observable across datasets generated independently of TCGA [18,19]. However, the adjusted GSE50081 estimate did not reach statistical significance, indicating that the magnitude and precision of the association vary across cohorts. These findings support reproducibility while also arguing against interpreting ADGRD1 as an established clinical biomarker on the basis of the present retrospective data alone.

The GSE30219 LUAD analysis also demonstrated the importance of evaluating survival-model assumptions. The proportional hazards assumption for ADGRD1 was violated in the standard continuous Cox model, suggesting that the relative association changed during follow-up. Nevertheless, the time-varying Cox model showed a favorable association throughout the clinically evaluated time points, although its magnitude attenuated over time. The RMST analysis, which does not rely on the proportional hazards assumption, independently demonstrated longer restricted mean survival in the ADGRD1-high group. Thus, the favorable LUAD association was not solely attributable to inappropriate use of a conventional proportional hazards model.

The molecular analyses also revealed histology-dependent transcriptional contexts. In LUAD, TP53 was the only prespecified molecular gene that remained significantly correlated with ADGRD1 after FDR correction. The previously suggested inverse association with EGFR expression was not reproduced using the harmonized GDC expression data, and the nominal BRAF association did not survive multiple-testing correction. In LUSC, ADGRD1 was inversely associated with CDKN2A, SOX2, and PIK3CA after FDR correction, whereas TP53 and NOTCH1 were not significantly correlated. Given the established molecular differences between LUAD and LUSC [3,4,5,6], these findings are compatible with different transcriptional contexts for ADGRD1 in the two histologies.

However, the molecular correlations were quantitatively modest, with the largest absolute correlation coefficient being 0.247 for SOX2 in LUSC. Expression–expression correlations should not be interpreted as evidence of direct regulation, pathway activation, or causal molecular interaction. Moreover, genomic mutation or copy-number status is biologically distinct from mRNA abundance [3,4]. Accordingly, the present data support subtype-specific transcriptional associations but do not establish a mechanistic relationship between ADGRD1 and TP53, CDKN2A, SOX2, PIK3CA, or other canonical driver pathways.

The LUSC findings warrant greater caution than the LUAD findings. Within TCGA-LUSC, the adverse association was statistically significant in the Kaplan–Meier analysis, univariate Cox model, clinically adjusted model, and continuous-expression models. Nevertheless, neither GSE30219 nor GSE50081 reproduced an adverse association. Both external point estimates were instead below 1 and nonsignificant, and the pooled estimate was also nonsignificant. Consequently, the present study does not establish a generalizable adverse prognostic role for ADGRD1 in LUSC. The TCGA-LUSC observation may reflect cohort-specific tumor composition, unmeasured clinical heterogeneity, residual confounding, or limited statistical precision in the external LUSC cohorts.

The clinicopathologic analyses likewise suggest that ADGRD1 cannot be interpreted simply as a surrogate for conventional disease severity. In LUAD, ADGRD1 expression was associated with pathologic stage, but most other clinical features and EGFR mutation status were not significantly different between expression groups. In LUSC, sex was significantly associated with expression, while overall pathologic stage, nodal status, and metastatic status were not. Importantly, multivariable survival associations persisted after adjustment for major measured clinicopathologic factors, particularly in LUAD.

Several limitations should be considered. First, this study was retrospective and based primarily on transcriptomic measurements; mRNA abundance does not necessarily correspond to ADGRD1 protein abundance, subcellular localization, receptor activation, or downstream signaling [7,8,9,10,13,14]. Second, although harmonized GDC expression values were used for the TCGA analyses, differences in assay platform and cohort composition remain between TCGA and the external GEO datasets. Third, the RFS variables were derived from investigator-curated TCGA analytic files and were not independently reconstructed from raw TCGA clinical records; standardized TCGA resources distinguish several related survival endpoints, including DFI and PFI [21]. Fourth, missing clinical and genomic information reduced the sample size of some complete-case analyses, particularly the EGFR-mutation-adjusted LUAD model. Mutation data for major driver genes, including EGFR, KRAS, and TP53, were also incomplete in a substantial proportion of the TCGA-LUAD cohort, limiting the statistical power of mutation-stratified analyses. Therefore, reliable associations between ADGRD1 expression and these driver mutations could not be established in the present study, and these exploratory analyses are not shown. Further studies with larger cohorts and more comprehensive genomic annotation are warranted to clarify the relationship between ADGRD1 expression and specific driver mutation profiles. Fifth, treatment information, comorbidities, performance status, and other potential prognostic determinants were not consistently available and may contribute to residual confounding. Finally, the adverse LUSC association lacked independent external confirmation, and no protein-level or functional experiments were performed.

Taken together, the present results support a histology-specific interpretation of ADGRD1 rather than its use as a uniform NSCLC marker. The favorable LUAD association was reproducible across multiple modeling approaches and independent datasets, whereas the adverse TCGA-LUSC association was not externally confirmed. Future studies integrating protein expression, genomic alterations, spatial or single-cell information, and functional experiments will be needed to determine the biological mechanisms underlying these divergent associations and to establish whether ADGRD1 provides clinically useful prognostic information beyond established variables.

## 5. Conclusions

ADGRD1 expression demonstrated distinct prognostic associations in LUAD and LUSC. Higher ADGRD1 expression was consistently associated with favorable survival in LUAD across median-dichotomized and continuous-expression analyses, clinical adjustment, EGFR-mutation sensitivity analysis, and independent GEO validation. In contrast, higher expression was associated with adverse OS in TCGA-LUSC, but this association was not reproduced in the external GEO cohorts. ADGRD1 also showed histology-specific but quantitatively modest transcriptional associations, with TP53 remaining significant in LUAD and CDKN2A, SOX2, and PIK3CA remaining significant in LUSC after FDR correction. These findings support further investigation of ADGRD1 as a subtype-specific prognostic marker, particularly in LUAD, while additional external validation and functional studies are required before mechanistic or clinical conclusions can be established.

## Figures and Tables

**Table 1:** Cox regression analyses of overall survival according to ADGRD1 expression in LUAD and LUSC.

Subtype	Model	ADGRD1 Scale	N/Events	HR (95% CI)	*p* Value
LUAD	Univariate	High vs. Low	492/179	0.542 (0.400–0.734)	<0.001
LUAD	Clinically adjusted	High vs. Low	483/177	0.543 (0.398–0.740)	<0.001
LUAD	Continuous	Per 1-SD increase	492/179	0.780 (0.668–0.909)	0.002
LUAD	Continuous adjusted	Per 1-SD increase	483/177	0.787 (0.673–0.919)	0.002
LUAD	Clinical + EGFR mutation sensitivity	High vs. Low	209/88	0.611 (0.391–0.956)	0.031
LUSC	Univariate	High vs. Low	488/212	1.444 (1.099–1.898)	0.008
LUSC	Clinically adjusted	High vs. Low	484/210	1.501 (1.137–1.982)	0.004
LUSC	Continuous	Per 1-SD increase	488/212	1.183 (1.041–1.345)	0.010
LUSC	Continuous adjusted	Per 1-SD increase	484/210	1.206 (1.062–1.369)	0.004

Clinically adjusted models included age group, sex, smoking status, and pathologic stage. The LUAD EGFR-mutation sensitivity model additionally included verified EGFR mutation status. HR, hazard ratio; CI, confidence interval; SD, standard deviation; LUAD, lung adenocarcinoma; LUSC, lung squamous cell carcinoma.

**Table 2:** Correlations between ADGRD1 expression and representative subtype-associated genes.

Subtype	Gene	N	Pearson r	*p* Value	BH-FDR q Value
LUAD	EGFR	493	−0.067	0.140	0.233
	KRAS	493	−0.011	0.800	0.800
	TP53	493	0.152	<0.001	0.004
	ALK	493	−0.037	0.409	0.512
	BRAF	493	0.092	0.041	0.103
LUSC	TP53	489	−0.047	0.299	0.374
	CDKN2A	489	−0.119	0.008	0.014
	SOX2	489	−0.247	<0.001	<0.001
	PIK3CA	489	−0.190	<0.001	<0.001
	NOTCH1	489	0.000	0.993	0.993

Pearson correlation coefficients were calculated using log2(FPKM-UQ + 1)-transformed gene-expression values obtained from the same GDC source. Benjamini–Hochberg FDR correction was applied separately to the five prespecified genes within each histologic subtype. FDR-adjusted q values < 0.05 were considered statistically significant.

**Table 3:** External validation of the association between ADGRD1 expression and overall survival in independent GEO cohorts.

Cohort	Subtype	N/Events	Unadjusted HR per 1-SD Increase (95% CI)	*p* Value	Adjusted HR per 1-SD Increase (95% CI)	*p* Value	Adjustment/Model
GSE30219	LUAD	83/43	0.588 (0.424–0.815)	0.001	0.559 (0.401–0.780)	<0.001	Age, sex, T stage, N stage
GSE50081	LUAD	128/52	0.744 (0.571–0.969)	0.028	0.790 (0.607–1.028)	0.079	Age, sex, pathologic stage
Pooled GEO (REML)	LUAD	—	0.675 (0.538–0.847)	<0.001	—	—	Random-effects meta-analysis
GSE30219	LUSC	61/43	0.912 (0.662–1.257)	0.574	0.857 (0.614–1.197)	0.366	Age, sex, T stage, N stage
GSE50081	LUSC	43/17	0.896 (0.498–1.612)	0.715	—	—	Not estimated
Pooled GEO (REML)	LUSC	—	0.909 (0.686–1.204)	0.504	—	—	Random-effects meta-analysis

Values are hazard ratios (HRs) for overall survival per one-standard-deviation (SD) increase in ADGRD1 expression unless otherwise indicated. Cohort-specific adjusted models used the clinical covariates available in each GEO dataset. Pooled estimates were obtained from the unadjusted continuous-expression Cox models using random-effects meta-analysis with restricted maximum likelihood estimation (REML). An adjusted model was not estimated for GSE50081 LUSC because an adequately specified covariate-adjusted model was unavailable. CI, confidence interval; LUAD, lung adenocarcinoma; LUSC, lung squamous cell carcinoma; REML, restricted maximum likelihood.

## Data Availability

Publicly available datasets were analyzed in this study. TCGA-LUAD and TCGA-LUSC clinical and gene-expression data are available through the National Cancer Institute Genomic Data Commons (GDC) Data Portal. The independent external-validation datasets are publicly available from the Gene Expression Omnibus (GEO) under accession numbers GSE30219 and GSE50081.

## Supplementary Materials

The following supporting information can be downloaded at https://www.mdpi.com/article/10.3390/cimb48090927/s1.
