# Supplementary material for: Subtype-Specific Prognostic and Molecular Significance of ADGRD1 in Lung Adenocarcinoma and Lung Squamous Cell Carcinoma
Source: Curr Issues Mol Biol. 2026 Sep 10;48(9):927. doi: 10.3390/cimb48090927 (PMC13605920; doi:10.3390/cimb48090927)
Supplement: Supplementary file 1 [file cimb-48-00927-s001.zip › cimb-4469129-supplementary.pdf]

Table S1. Clinicopathologic characteristics according to ADGRD1 expression in TCGA-LUAD and TCGA-LUSC.

Variables		LUAD			LUSC		
		High	Low	P	High	Low	P
Age	≤65	115 (46.9%)	121 (49.2%)	0.618	88 (36.1%)	101 (41.4%)	0.227
	>65	130 (53.1%)	125 (50.8%)		156 (63.9%)	143 (58.6%)	
Gender	F	140 (56.9%)	127 (51.6%)	0.239	74 (30.3%)	52 (21.3%)	**0.023**
	M	106 (43.1%)	119 (48.4%)		170 (69.7%)	192 (78.7%)	
Smoking	Yes	173 (70.3%)	162 (65.6%)	0.260	212 (86.9%)	201 (82.0%)	0.139
	No	73 (29.7%)	85 (34.4%)		32 (13.1%)	44 (18.0%)	
T stage	T1	91 (37.4%)	75 (30.5%)		66 (27.0%)	43 (17.6%)	
	T2	126 (51.9%)	134 (54.5%)	0.285	133 (54.5%)	153 (62.7%)	0.067
	T3	18 (7.4%)	27 (11.0%)		32 (13.1%)	38 (15.6%)	
	T4	8 (3.3%)	10 (4.1%)		13 (5.3%)	10 (4.1%)	
N stage	N0	167 (71.1%)	151 (61.4%)		157 (65.7%)	154 (63.4%)	
	N1	38 (16.2%)	55 (22.4%)	0.075	62 (25.9%)	64 (26.3%)	0.344
	N2	30 (12.8%)	38 (15.4%)		16 (6.7%)	24 (9.9%)	
	N3	0 (0.0%)	2 (0.8%)		4 (1.7%)	1 (0.4%)	
M stage	M0	160 (91.4%)	164 (94.8%)	0.215	197 (98.0%)	204 (98.6%)	0.721
	M1	15 (8.6%)	9 (5.2%)		4 (2.0%)	3 (1.4%)	
Pathologic stage	I	142 (59.4%)	122 (49.8%)	**0.043**	123 (50.8%)	115 (47.5%)	0.696
	II	49 (20.5%)	68 (27.8%)		72 (29.8%)	84 (34.7%)	

	III	33 (13.8%)	46 (18.8%)		43 (17.8%)	40 (16.5%)	
	IV	15 (6.3%)	9 (3.7%)		4 (1.7%)	3 (1.2%)	
EGFR mutation	Mutant	13 (12.1%)	16 (15.1%)	0.531	—	—	—
	Wild-type	94 (87.9%)	90 (84.9%)		—	—	

Data are presented as n (%). Percentages were calculated using available observations for each variable; therefore, denominators may differ because of missing clinical information. Pearson's chi-square test was used when expected cell-count assumptions were adequately satisfied. Fisher's exact test or the Fisher–Freeman–Halton test with Monte-Carlo estimation was used for sparse contingency tables as appropriate. Verified EGFR mutation-positive/wild-type information was available only for a subset of LUAD patients and was not available in the supplied LUSC dataset. LUAD, lung adenocarcinoma; LUSC, lung squamous cell carcinoma.

Table S2. Sensitivity analyses for the proportional-hazards assumption in GSE30219 LUAD.

**Analysis**	**Measure**	**Estimate**	**95% CI**	**P value**
Standard continuous Cox	HR per 1-SD increase	0.588	0.424–0.815	0.001
Schoenfeld residual test	PH-assumption test for ADGRD1	—	—	0.027
Time-varying Cox	ADGRD1 × log(1 + time/12) interaction	β = 0.575	—	0.020
Time-varying Cox	HR at 12 months	0.382	0.230–0.635	—
Time-varying Cox	HR at 36 months	0.569	0.407–0.795	—
Time-varying Cox	HR at 60 months	0.719	0.500–1.033	—
Adjusted time-varying Cox	ADGRD1 × log(1 + time/12) interaction	β = 0.541	—	0.031
Adjusted time-varying Cox	HR at 12 months	0.374	0.223–0.626	—
Adjusted time-varying Cox	HR at 36 months	0.544	0.386–0.766	—
Adjusted time-varying Cox	HR at 60 months	0.677	0.467–0.981	—

60-month RMST	ADGRD1-low group	39.80 months	—	—
60-month RMST	ADGRD1-high group	54.79 months	—	—
60-month RMST	Difference, High − Low	14.98 months	7.46–22.50 months	<0.001

The standard continuous Cox model showed evidence of non-proportional hazards for ADGRD1 based on the Schoenfeld residual test. A time-varying coefficient model was therefore fitted using an ADGRD1 × log(1 + time/12) interaction. The adjusted time-varying Cox model included age, sex, T stage, and N stage. Restricted mean survival time (RMST) was calculated through 60 months using the median-dichotomized ADGRD1 groups and does not require the proportional-hazards assumption. Dashes indicate quantities not directly estimated in the corresponding analysis. CI, confidence interval; HR, hazard ratio; LUAD, lung adenocarcinoma; PH, proportional hazards; RMST, restricted mean survival time; SD, standard deviation.

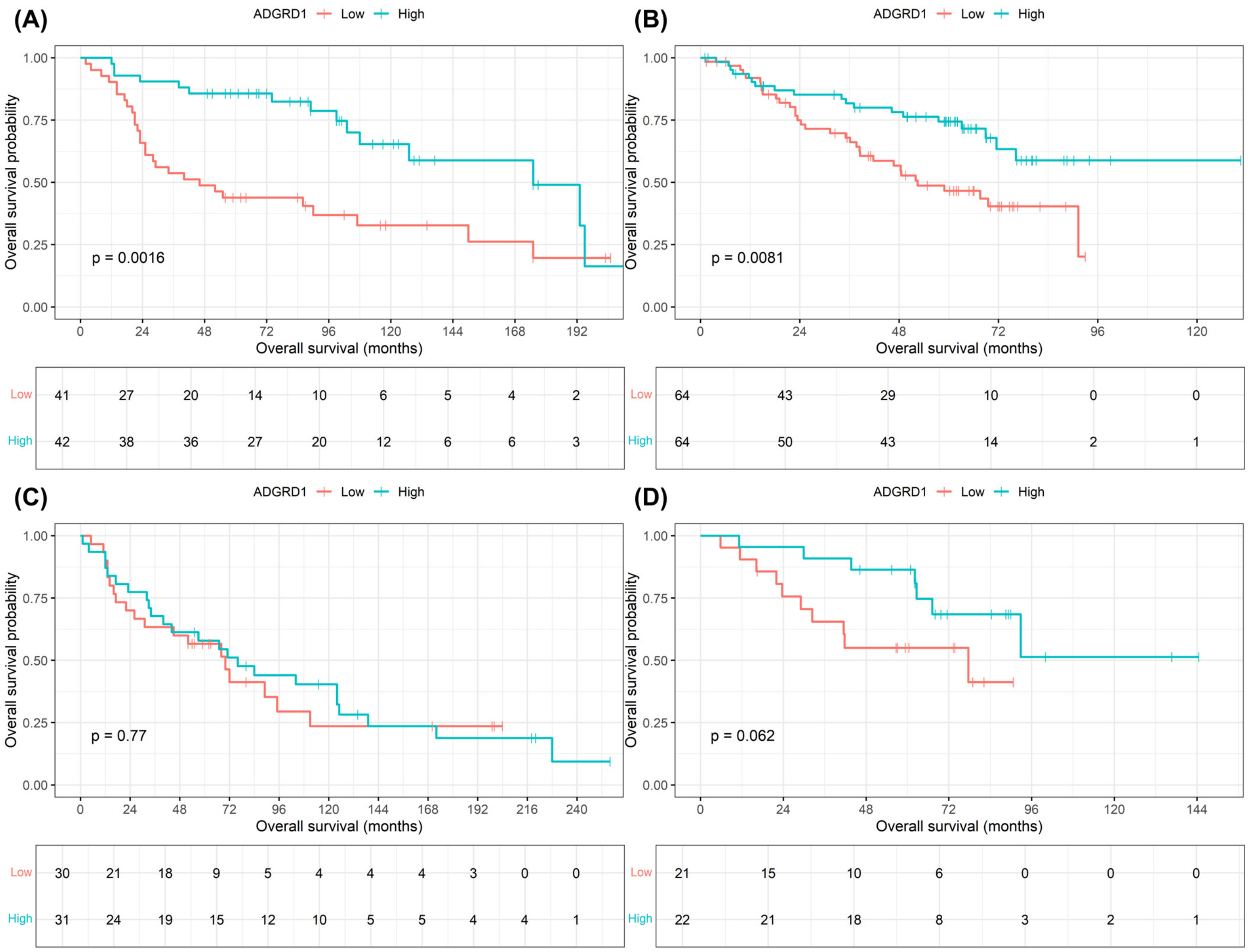

Figure S1. Independent GEO validation of the prognostic association of ADGRD1 expression in LUAD and LUSC. (A) GSE30219 LUAD; (B) GSE50081 LUAD; (C) GSE30219 LUSC; and (D) GSE50081 LUSC. Patients were classified into ADGRD1-high and ADGRD1-low groups using the cohort- and subtype-

specific median expression value. Overall survival was compared using the log-rank test. Numbers at risk are shown below the survival curves. Corresponding continuous-expression Cox regression results are presented in Table 3.

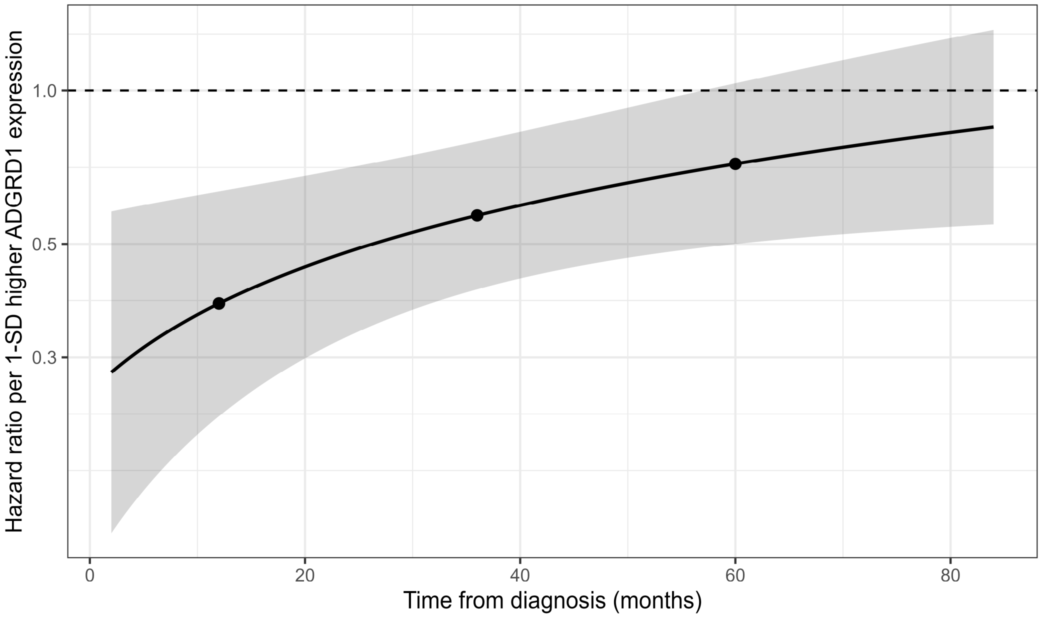

Figure S2. Time-varying prognostic association of ADGRD1 in the GSE30219 LUAD cohort. The curve represents the estimated time-varying hazard ratio for overall survival associated with higher ADGRD1 expression, and the shaded area indicates the 95% confidence interval. The horizontal reference line represents an HR of 1. Because the proportional-hazards assumption was not satisfied in the standard continuous Cox model, a time-varying coefficient model was fitted. Detailed model estimates and complementary restricted mean survival time analyses are provided in Table S2.
